# Variation in *LPA* Is Associated with Lp(a) Levels in Three Populations from the Third National Health and Nutrition Examination Survey

**DOI:** 10.1371/journal.pone.0016604

**Published:** 2011-01-28

**Authors:** Logan Dumitrescu, Kimberly Glenn, Kristin Brown-Gentry, Cynthia Shephard, Michelle Wong, Mark J. Rieder, Joshua D. Smith, Deborah A. Nickerson, Dana C. Crawford

**Affiliations:** 1 Center for Human Genetics Research, Vanderbilt University, Nashville, Tennessee, United States of America; 2 Department of Molecular Physiology and Biophysics, Vanderbilt University, Nashville, Tennessee, United States of America; 3 Department of Genome Sciences, University of Washington, Seattle, Washington, United States of America; Innsbruck Medical University, Austria

## Abstract

The distribution of lipoprotein(a) [Lp(a)] levels can differ dramatically across diverse racial/ethnic populations. The extent to which genetic variation in *LPA* can explain these differences is not fully understood. To explore this, 19 *LPA* tagSNPs were genotyped in 7,159 participants from the Third National Health and Nutrition Examination Survey (NHANES III). NHANES III is a diverse population-based survey with DNA samples linked to hundreds of quantitative traits, including serum Lp(a). Tests of association between *LPA* variants and transformed Lp(a) levels were performed across the three different NHANES subpopulations (non-Hispanic whites, non-Hispanic blacks, and Mexican Americans). At a significance threshold of p<0.0001, 15 of the 19 SNPs tested were strongly associated with Lp(a) levels in at least one subpopulation, six in at least two subpopulations, and none in all three subpopulations. In non-Hispanic whites, three variants were associated with Lp(a) levels, including previously known rs6919246 (p = 1.18×10^−30^). Additionally, 12 and 6 variants had significant associations in non-Hispanic blacks and Mexican Americans, respectively. The additive effects of these associated alleles explained up to 11% of the variance observed for Lp(a) levels in the different racial/ethnic populations. The findings reported here replicate previous candidate gene and genome-wide association studies for Lp(a) levels in European-descent populations and extend these findings to other populations. While we demonstrate that *LPA* is an important contributor to Lp(a) levels regardless of race/ethnicity, the lack of generalization of associations across all subpopulations suggests that specific *LPA* variants may be contributing to the observed Lp(a) between-population variance.

## Introduction

Lipoprotein (a) [Lp(a)] levels have long been recognized as an independent risk factor for coronary artery disease (CAD)[1]–[3]. However, Lp(a) concentrations and their relationship with cardiovascular disease vary across races/ethnicities. The most notable example of this discrepancy is observed between populations of European- and African-decent. While the mean Lp(a) level is two- to threefold higher in blacks relative to whites[4], [5], elevated plasma Lp(a) levels have been reported to be associated with CAD in whites but have not been clearly demonstrated in blacks[6]–[10].

The epidemiology of Lp(a) in other US racial/ethnic populations, such as Mexican Americans, is not as well documented and often inconsistent. For example, compared to non-Hispanic whites, studies have shown Mexican Americans to have both higher[11] and lower[12] mean Lp(a) levels. The underlying cause(s) for these between-population differences has not been fully determined; however, there is evidence for the role of multiple, population-specific alleles in *LPA* [13], the gene that encodes for apolipoprotein(a) [apo(a)], which when bound to apolipoprotein B-100 and a low density lipoprotein (LDL)-like particle forms Lp(a).

Lp(a) levels not only vary dramatically across populations, they also have a remarkable inter-individual variability that ranges from barely detectable to greater than 250 nmol/l[14]. This inter-individual variability has a substantial genetic component. It has been determined that the apolipoprotein(a) gene is the major contributor to Lp(a) levels, accounting for more than 90% of the variance for that trait in European Americans[15].

Two types of genetic variants in *LPA* have been associated with Lp(a) levels: variations in the number of copies of the kringle IV-2 repeat and single nucleotide polymorphisms (SNPs). It has been estimated that the kringle IV-2 repeat alone explains 61–69% of the variability observed in Lp(a) levels in populations of European ancestry[15], [16]. In contrast, the kringle repeat appears to explain less of the variability (19–44%) in populations of African descent[17]–[19] and Mexican Americans (22–48%)[20], [21]. While the kringle IV-2 repeat polymorphism accounts for a large percentage of the variability of Lp(a) levels, the remaining variance has yet to be explained.

Recent studies have identified common SNPs in *LPA* as strongly associated with Lp(a) levels, explaining up to 36% of the trait variance in populations of European-descent[22]–[24]. While several studies have indicated certain SNPs are in substantial linkage disequilibrium (LD) with the kringle IV-2 repeat polymorphism[22], [23], evidence also exists that some SNPs are in relatively little LD with copy number variation in *LPA* [25] and may be independent contributors to Lp(a) levels. A recent genome-wide association study performed in a Hutterite population with kringle IV-2 repeat polymorphism data identified a SNP associated with Lp(a) levels independent of the kringle repeat, supporting the assumption that some common SNPs in *LPA* are independent of the kringle repeat polymorphisms (i.e., not in linkage disequilibrium)[24].

To date, relatively few studies have examined associations between *LPA* common SNPs and Lp(a) levels across multiple, diverse populations and no study has characterized the same panel of *LPA* common SNPs in populations of European-, African-, and Mexican-descent. To better characterize this genotype-phenotype relationship in more diverse populations, we have genotyped 19 European American and African American *LPA* tagSNPs in 7,159 participants from the Third National Health and Nutrition Examination Survey (NHANES III). NHANES III is a diverse, population-based cohort representing Americans of European-, African-, and Mexican-descent[26]. We report the significant association of *LPA* SNPs and Lp(a) levels in this diverse cohort and estimate the proportion of Lp(a) variance explained by these genetic variants.

## Results

### Population Characteristics

Characteristics of the NHANES III study participants are shown in Table 1. Genetic NHANES III included 2,631 non-Hispanic whites, 2,108 non-Hispanic blacks, and 2,073 Mexican Americans. As expected[27], the mean Lp(a) level in non-Hispanic blacks was 43.4 mg/dL (SD, 32.8 mg/dL), a twofold increase compared to non-Hispanic whites and a three-fold increase compared to Mexican Americans. Mexican Americans had significantly lower mean Lp(a) levels compared to whites (p<0.0001). Body mass index (BMI) was similar across all three populations (p = 0.093). Demographic variables age and sex, along with other blood lipid traits LDL-C, HDL-C, and triglycerides, differed significantly (p<0.0001) across populations.

**Table 1:** NHANES III study population characteristics.

Variable	Non-Hispanic Whites(n = 2,631)	Non-Hispanic Blacks(n = 2,108)	Mexican Americans(n = 2,073)	P-value
Males, %	40.0%	42.6%	49.4%	<0.0001
Age (yr)	50.2 (22.3)	36.0 (18.3)	37.1 (18.7)	<0.0001
BMI (kg/m^2^)	26.3 (5.6)	27.3 (6.8)	27.0 (5.6)	0.093
Lp(a) (mg/dL)	20.3 (24.1)	43.4 (32.8)	14.9 (8.5)	<0.0001
HDL-C (mg/dL)	50.2 (15.6)	53.8 (16.4)	48.0 (13.1)	0.112
LDL-C (mg/dL)	127.0 (38.0)	118.8 (39.5)	116.3 (34.1)	<0.0001
TG (mg/dL)	147.63 (116.8)	108.8 (79.9)	154.1 (121.2)	<0.0001

Study characteristics are shown for participants greater than 18 years of age who had non-missing Lp(a) levels. Samples sizes shown are the DNA samples available in Genetic NHANES III for each subpopulation. Values are shown as unweighted mean (SD). BMI, body mass index; Lp(a), lipoprotein(a); HDL-C, high-density lipoprotein cholesterol; LDL-C, low-density lipoprotein cholesterol; TG, triglycerides. P-values are based on one-way unweighted ANOVA.

TagSNP allele frequencies are presented in Supplementary Table S1, by population. We calculated the Pearson correlation coefficient (r) between each of the three populations. Not surprisingly[28], [29], *LPA* allele frequencies observed in non-Hispanic whites were highly correlated with allele frequencies observed in Mexican Americans (r = 0.80). Also as expected[28]–[30], we observed weaker correlation between allele frequencies in non-Hispanic blacks compared with non-Hispanic whites (r = 0.60) and Mexican Americans (r = 0.48). Furthermore, compared with non-Hispanic whites, the proportion of SNPs that differed in allele frequency by more than ±0.10 was smaller in Mexican Americans (2/19 SNPs; 11%) than in blacks (11/19 SNPs; 58%).

We also compared the allele frequencies of these *LPA* SNPs in NHANES III to those in HapMap[31], [32] (Supplementary Table S2). Among the 12 *LPA* SNPs that overlapped this dataset and HapMap, we observed extremely high correlations (r≥0.99) in allele frequencies between non-Hispanic whites and HapMap CEU (US individuals of northern and western European ancestry) and between non-Hispanic blacks and both HapMap YRI (Yoruba from West Africa) and ASW (individuals with African ancestry from the Southwest USA). Mexican American allele frequencies were also very similar (r = 0.93) to those of HapMap MEX (individuals with Mexican ancestry in Los Angeles, California). Because Mexican Americans are a historically admixed population, a comparison with HapMap Asian populations was performed. The correlation between NHANES Mexican Americans and HapMap Han Chinese (HCB) and Japanese (JPT) was 0.77 and 0.78, respectively.

Haplotype frequencies were inferred for the 19 tagSNPs in *LPA* by NHANES III subpopulation. We observed eight common haplotypes (frequency >5%) in at least one subpopulation (Supplementary Table S3). While two haplotypes (#1 and #2) were common across all three populations, the remaining haplotypes were either common only to non-Hispanic blacks (#7 and #8), only non-Hispanic whites (#6), or shared between whites and Mexican Americans (#3, #4, #5). As expected[33], the majority of chromosomes from non-Hispanic whites (71.5%) and Mexican Americans (72.6%) were represented by common haplotypes inferred from *LPA* tagSNPs. Only approximately half of the chromosomes from non-Hispanics blacks (55.7%) were represented by common haplotypes, and the remaining half are scattered across rare haplotypes.

### *LPA* SNP associations with Lp(a) levels

**Figure 1:**
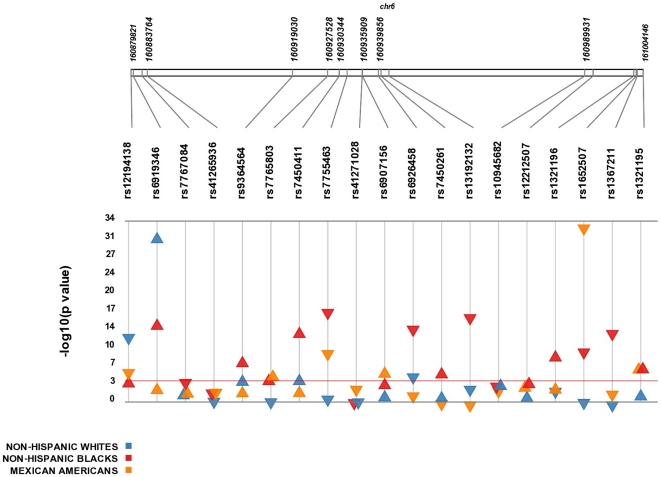
Overview of association results between *LPA* SNPs and Lp(a) levels. Plot showing the significance of all single-SNP associations with transformed Lp(a) levels. All results are unweighted adjusted for age and sex and are stratified by race/ethnicity. SNPs are plotted on top along the *x* axis in order from 5′ to 3′, and association with Lp(a) is indicated on the *y* axis (as −log_10_ p-value). Red line indicates p-value of 1×10^−4^. Direction of the triangle indicates direction of effect (β-coefficient).

Each SNP was tested for an association with transformed Lp(a) levels. Results from this analysis are presented in Figure 1 and Table 2. After adjusting for age and sex, 15 of the 19 SNPs tested were significantly associated with Lp(a) levels in at least one subpopulation at p<0.0001, meeting the standard Bonferroni p-value threshold for multiple testing. Among non-Hispanic whites, we confirmed previous evidence of a strong association with rs6919346 (p = 1.2×10^−30^)[22], [24], which explained approximately 6% of the trait variance (R^2^ = 0.057) in our dataset. We also identified two novel associations with rs6926458 and rs12194138 (p = 5.3×10^−6^ and 2.1×10^−13^, respectively). To evaluate the combined effects of significantly associated variants, we calculated a continuous Genetic Risk Score (GRS) for each participant based on his or her total number of risk (i.e. Lp(a) increasing) alleles at each associated SNP. Based on the GRS, the additive effect of rs6919646, rs6926458, and rs12194138 explained 7% of the variation in transformed Lp(a) levels in non-Hispanic whites (Table 3).

**Table 2:** Associations between *LPA* SNPs and Lp(a) levels.

SNPs	Non-Hispanic Whitesn = 2,397			Non-Hispanic Blacksn = 1,711			Mexican Americansn = 1,749		
	β(95% CI)	R^2^	P-value	β(95% CI)	R^2^	P-value	β(95% CI)	R^2^	P-value
**rs1321196**	−0.13(−0.22, −0.05)	0.0036	0.0026	0.21(0.14, 0.28)	0.0193	**1.88×10^−8^**	0.12(0.02, 0.22)	0.0031	0.0204
**rs1321195**	0.05(−0.07, 0.18)	0.0003	0.3863	0.53(0.31, 0.75)	0.0123	**3.03×10^−6^**	0.40(0.23, 0.56)	0.0127	**3.49×10^−6^**
**rs1367211**	−0.004(−0.10, 0.09)	0.0000	0.9330	−0.27(−0.34, −0.20)	0.0345	**3.67×10^−14^**	−0.15(−0.26, 0.04)	0.0039	0.0092
**rs1652507**	−0.05(−0.16, 0.05)	0.0005	0.3295	−0.45(−0.59, −0.32)	0.0247	**1.06×10^−10^**	−0.54(−0.63, −0.46)	0.0858	**5.44×10^−34^**
**rs6907156**	0.21(−0.51, 0.92)	0.0001	0.5722	0.15(0.05, 0.25)	0.0053	0.0031	0.73(0.39, 1.06)	0.0103	**2.14×10^−5^**
**rs6919346**	0.61(0.51, 0.71)	0.0565	**1.18×10^−30^**	0.75(0.56, 0.94)	0.0344	**2.16×10^−14^**	0.18(0.02, 0.33)	0.0029	0.0248
**rs6926458**	−0.23(−0.32, −0.13)	0.0087	**5.29×10^−6^**	−0.45(−0.57, −0.34)	0.0364	**5.90×10^−15^**	−0.15(−0.28, 0.03)	0.0032	0.0189
**rs7755463**	−0.47(−0.99, 0.06)	0.0014	0.0823	−0.33(−0.40, −0.25)	0.0449	**4.01×10^−18^**	−0.89(−1.16. −0.62)	0.0230	**2.14×10^−10^**
**rs7767084**	0.07(−0.05, 0.18)	0.0006	0.2467	−0.41(−0.61, −0.21)	0.0097	**5.88×10^−5^**	0.10(−0.03, 0.23)	0.0015	0.1187
**rs9364564**	0.19(0.08, 0.29)	0.0049	0.0007	0.33(0.21, 0.46)	0.0156	**2.29×10^−7^**	0.11(−0.02, 0.24)	0.0016	0.1053
**rs12212507**	0.03(−0.15, 0.21)	0.0001	0.7633	0.63(0.23, 1.03)	0.0063	0.0019	0.57(0.14, 1.00)	0.0037	0.0093
**rs13192132**	−0.14(−0.23, −0.06)	0.0043	0.0011	−0.43(−0.53, −0.33)	0.0425	**3.85×10^−17^**	−0.003(−0.11, 0.10)	0.0000	0.9508
**rs10945682**	0.12(0.04, 0.21)	0.0033	0.0041	−0.14(−0.21, −0.06)	0.0079	0.0003	−0.16(−0.26, −0.06)	0.0055	0.0022
**rs12194138**	−0.41(−0.52, −0.30)	0.0229	**2.05×10^−13^**	0.34(0.12, 0.56)	0.0020	0.0014	−0.47(−0.66, −0.28)	0.0149	**7.97×10^−7^**
**rs7450261**	0.10(−1.29, 1.48)	0.0000	0.8907	0.35(0.18, 0.51)	0.0108	**2.84×10^−5^**	−0.33(−1.34, 0.68)	0.0003	0.5176
**rs7450411**	0.19(0.08, 0.30)	0.0051	0.0005	0.37(0.27, 0.47)	0.0308	**7.37×10^−13^**	0.12(−0.02, 0.25)	0.0017	0.0910
**rs7765803**	−0.05(−0.14, 0.04)	0.0005	0.2471	0.13(0.06, 0.20)	0.0071	0.0005	0.21(0.10, 0.31)	0.0089	**8.54×10^−5^**
**rs41265936**	−0.80(−2.04, 0.44)	0.0007	0.2037	−0.22(−0.37, −0.06)	0.0047	0.0054	−0.88(−1.48, −0.28)	0.0046	0.0039
**rs41271028**	−0.45(−1.22, 0.32)	0.0006	0.2556	−0.06(−0.18, 0.07)	0.0007	0.3608	−0.66(−1.06, −0.27)	0.0061	0.0011

The association of *LPA* SNPs with log transformed Lp(a) levels is shown by a regression coefficient (beta, β) and 95% confidence interval (CI) for each SNP, adjusted for age and sex. Measures of variance explained (R^2^) are provided for each SNP based on unadjusted regressions. Significant associations (P-value<0.0001) are in bold.

**Table 3:** Additive effects of *LPA* risk alleles on Lp(a) levels.

	Non-Hispanic Whites	Non-Hispanic Blacks	Mexican Americans
Total n	2269	1605	1665
No. SNPs used in GRS	3	12	6
β (95% CI)	0.36 (0.31–0.42)	0.11 (0.09–0.13)	0.34 (0.29–0.39)
P-value	<10^−35^	<10^−34^	<10^−41^
R^2^	0.07	0.09	0.11

The amount of variance explained (R^2^) in transformed Lp(a) levels by the Genetic Risk Score (GRS) is displayed, along with the regression coefficient (beta, β) and 95% confidence interval (CI) for each association.

Mexican Americans had twice the number of significant associations compared with non-Hispanic whites, with six SNPs associated with transformed Lp(a) levels at p<0.0001. One SNP in particular, rs1652507, was strongly associated at p = 5.44×10^−34^ and had the largest effect size of all the associations (R^2^ = 0.086). Two of the six associated SNPs (rs1321195 and rs7765803) have previously been associated with Lp(a) in a cohort of Europeans[22]. The joint effect of all six associated SNPs, as measured by the GRS, explained 11% of the variance in Lp(a) trait distribution observed in Mexican Americans.

Of the three subpopulations, non-Hispanic blacks had the greatest number of significant associations at p<0.0001 with 12 SNPs. Each associated SNP contributed 1% to 4.5% of the trait variance, with the additive effect of the SNPs contributing up to 9% of the total variance in Lp(a) levels. Five of the 12 associated SNPs (rs1321195, rs1652507, rs6919346, rs6926458, and rs7755463) were also associated in one of the two other racial/ethnic groups, non-Hispanic whites or Mexican Americans, and the directions of the effect (beta) were consistent across the associated subpopulations.

### *LPA* risk allele distribution

**Figure 2:**
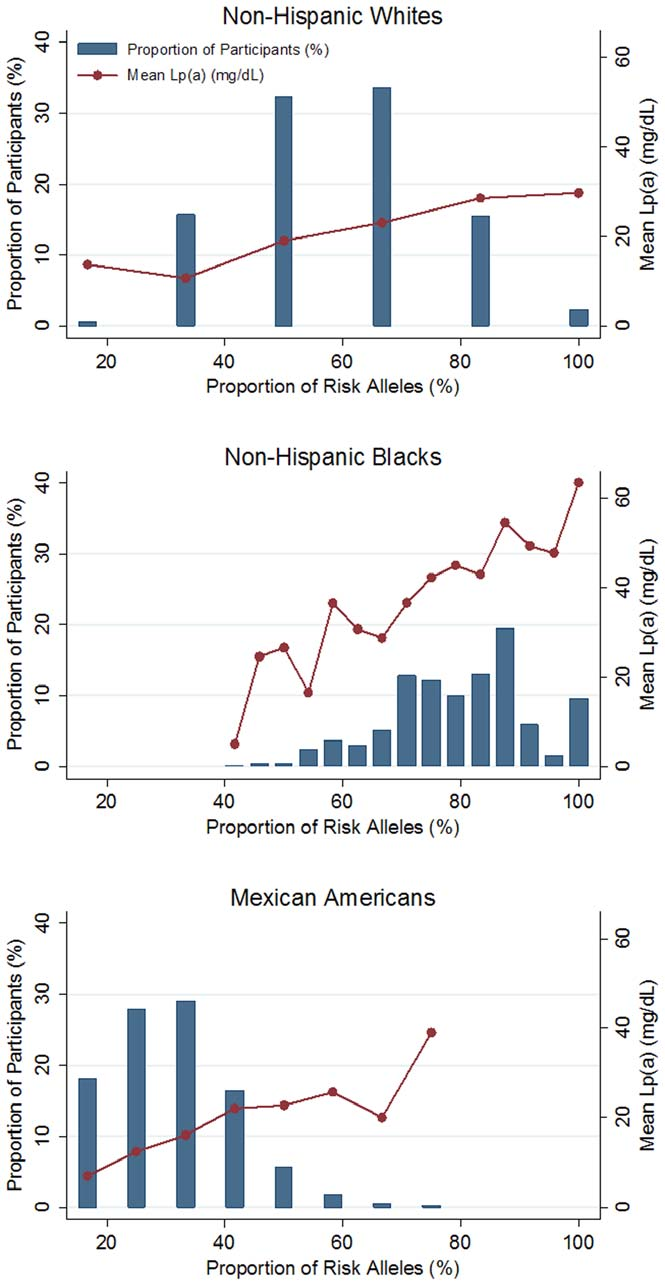
Distribution of *LPA* risk alleles in non-Hispanic whites, non-Hispanic blacks, and Mexican Americans. Plots showing the frequency distributions of the proportion of risk alleles in the three NHANES III subpopulations. Proportion of risk alleles was calculated by dividing the total number of *LPA* risk alleles (i.e. the GRS, see Materials and Methods) by the total number of possible risk alleles in each population, multiplied by 100%. Mean Lp(a) values are also plotted for each corresponding proportion.

The proportion of risk alleles (i.e. the total number of risk alleles divided by the number of risk alleles possible) was examined across all NHANES subpopulations (Figure 2). In general, the distributions differed greatly among non-Hispanic whites, non-Hispanic blacks, and Mexican Americans. In non-Hispanic whites, the proportion of risk alleles followed a normal distribution and the average (mean) number of risk alleles was 3.5 out of the possible six risk alleles (58.3%). In contrast, the distribution of risk alleles was skewed to the left in non-Hispanic blacks and to the right in Mexican Americans (Figure 2). The average number of risk alleles in non-Hispanic black participants was 17 out of 24 possible risk alleles (70.8%) while the average number in Mexican American participants was 5.5 out of the 12 possible risk alleles (45.8%). Overall, non-Hispanic blacks had the largest genetic burden of all three subpopulations defined by these alleles, with 99.0% percent of participants possessing greater than 50% of the possible risk alleles. This genetic burden is significantly greater than that carried by non-Hispanic whites (51.4%, p<0.001) or Mexican Americans (2.7%, p<0.001). Figure 2 also illustrates mean Lp(a) levels in participants with various proportions of risk alleles. As expected, mean Lp(a) is higher in participants with a greater proportion of risk alleles, again reflecting the role that these variants may play in contributing to both between- and within-population Lp(a) trait variation.

## Discussion

In this study, we identified several variants in the *LPA* gene that are strongly associated with Lp(a) levels in a diverse epidemiologic study. More specifically, three SNPs in non-Hispanic whites, twelve SNPs in non-Hispanic blacks, and six SNPs in Mexican Americans were strongly associated at p<0.0001. While no single *LPA* variant was significantly associated in all three racial/ethnic groups, six SNPs were significantly associated in two subpopulations and the directions of effects were consistent.

Most previously published studies characterizing the relationship between Lp(a) and *LPA* have focused on the effects of the kringle IV-2 copy number polymorphism. More recently, a genome-wide association study in Hutterites identified one SNP in *LPA* (rs6919346) that associated with Lp(a) levels, independent of kringle IV-2 copy number[24]. Subsequent studies have found this variant to be independently associated with increased Lp(a) levels in European Caucasians[22], [23] and South Asians and Chinese[23]. In our study, the same allele (G) was also strongly associated with increased trait levels not only in non-Hispanic whites (β = 0.61, p = 1.18×10^−30^) but also in non-Hispanic blacks (β = 0.75, p = 2.16×10^−14^). In contrast, the association in Mexican Americans was much less robust (p = 0.02), but the effect trended in the same direction (β = 0.18). This intronic tagSNP is not in linkage disequilibrium (LD) with any genotyped SNP (Supplementary Figure S1), nor with the kringle IV repeat[25]. As others have suggested[24], rs6919346 may be tagging the causal variant or, due to the fact that it resides in a CRE-binding site, may play a role in gene expression.

It is interesting to note that while we did replicate the association between Lp(a) levels and rs6919346, we did not necessarily replicate the associations reported recently for rs10945682 and rs7765803[23]. *LPA* rs10945682 was not associated with Lp(a) levels in NHANES III at the significant threshold of p<0.0001 (Table 2). Furthermore, while the direction of effect in non-Hispanic whites was consistent with that observed for Europeans and Asians studied by Lanktree et al (taking into account the coded allele), the direction of effect was opposite in the non-Hispanic black and Mexican American subpopulations in NHANES III. *LPA* rs7765803 was not associated with Lp(a) levels in non-Hispanic whites (p = 0.2471) while it was strongly associated in European and Asian populations in Lanktree et al. Finally, the data reported here are not consistent with the linkage disequilibrium data reported by Lanktree et al. *LPA* rs10945682 and rs6919346 are reported to be in the same linkage disequilibrium block[23] but the LD calculated in our non-Hispanic white samples and in HapMap CEU suggests there is little LD (r^2^ = 0.06 and 0.03 in non-Hispanic whites and CEU, respectively) between the two SNPs. It is possible that this discrepancy can be explained by unidentified population substructure or by the use of different LD measures, but this is unclear from the literature and requires further investigation.

As alluded to above, the relationship between *LPA* tagSNPs and Lp(a) levels may represent a direct (i.e. causal) or indirect (i.e. proxy for true causal variant) relationship. The latter situation most likely applies to the majority of SNPs genotyped in this study. Of the 19 *LPA* SNPs, 17 are located in introns, and the two nonsynonymous SNPs (rs7765803 and rs41265936, Supplementary Table S1) are not predicted to alter protein function using SIFT[34]. Additional studies are needed to determine if these variants regulate *LPA* expression *in vivo*. However, since apo(a) is present only in humans, Old World primates, and the hedgehog, resources for these studies are limited to transgenic mice and rabbits as models[35].

In an attempt to evaluate the joint effect of significantly associated variants, a genetic risk score (GRS) was calculated. Based on this GRS, these variants together explained 7%, 9%, and 11% of the variance in Lp(a) levels in non-Hispanic whites, non-Hispanic blacks, and Mexican Americans, respectively. In comparison to the effect attributed to the kringle repeat region based on previous studies[15], [16], [20], [21], [28], [36]–[38], the effect of these SNPs is considerably small.

This study has several strengths and limitations. The greatest strength is the use of a large and diverse population. While there have been several studies of *LPA* SNPs and its association with Lp(a) that have included both European and African descent populations, no single study, to our knowledge, has also included Mexican Americans genotyped for the same *LPA* SNPs. This latter point cannot be under emphasized as the Hispanic or Latino population is the fastest growing minority population in the United States yet remains relatively underrepresented in genetic association studies[39].

A limitation is that the method of measuring serum Lp(a) levels in NHANES III does not account for apo(a) isoform size. While accurate measurement of apo(a) isoform is ideal, the reliability of the Lp(a) measurement used here has been adequately demonstrated[26]. Furthermore, there is no generally accepted laboratory procedure or national standardization program for Lp(a) measurement, which may help to explain the lack of generalizabilty across studies[14].

A second major limitation is that NHANES III does not have data on kringle repeat size for each participant. Several methods are used to measure kringle repeat size such as Southern blot[28] and quantitative PCR[40], neither of which can be used in NHANES III DNA samples given investigators are aliquoted limited amounts of DNA from crude cell lysates. Without these data, it is unclear if the associations between *LPA* SNPs and Lp(a) levels reported here are independent of the KIV-2 copy number variant, which has a well-established, large effect on Lp(a) levels.

The amount of linkage disequilibrium, or lack thereof, between the KIV-2 region and other *LPA* variants is a controversial issue. Previous studies have reported strong LD between the KIV-2 alleles and SNPs in or around *LPA* [22], [41]–[43]. In contrast, additional studies indicate the lack of strong LD[24], [28]. More specifically, the tagSNPs genotyped in this study had been selected from a previous study[28] that provided data on kringle IV-2 repeat size, and no strong LD (r^2^>0.80) was found for any of the SNPs tested[28]. However, there was moderate LD (r^2^ = 0.45 in European American and r^2^ = 0.57 in African American samples) between kringle repeat sizes 10 and 14 and *LPA* SNPs 74970 and rs41271028, respectively[28]. *LPA* 74970 was not genotyped here. *LPA* rs41271028 was genotyped here but was not significantly associated with Lp(a) levels in any of the three subpopulations after correction for multiple testing (Table 2). Thus, the tagSNPs genotyped here and significantly associated with Lp(a) levels after correction for multiple testing are not in high or moderate LD with specific kringle repeat sizes examined in the original dataset reported by Crawford et al[28]. Further studies are needed in NHANES and other large datasets to characterize the full spectrum of *LPA* genetic variation and its impact on Lp(a) levels in diverse populations.

Another limitation of this study is that only approximately 30–35% of the LDSelect “bins” for European Americans and African Americans are represented by tagSNPs as many *LPA* SNPs failed assay design or genotyping in NHANES III. And, tagSNPs selection was limited to common variation, leaving rarer variation such as *LPA* rs10455872 (<5% MAF) untested. Thus, much of the genetic variation in *LPA* and its association with Lp(a) levels in these populations remains to be explored. Furthermore, tagSNPs were not selected specifically for the Mexican American subpopulation. At the time of tagSNPs selection, HapMap 3 Mexican American samples were not available at the time, and it was unclear which populations should be used for tagSNPs selection to adequately represent this admixed population. It is important to note that, however, that while our tagSNPs selection process may have been biased for populations of European- and African-descent, the allele frequencies observed in NHANES III Mexican Americans were very similar to that of non-Hispanic whites. Furthermore, our lack of Mexican American specific tagSNPs does not undermine the observation that there is an excess of significant variants associated only in non-Hispanic blacks compared to non-Hispanic whites.

Because of these strengths, and despite these limitations, we have taken an important step in understanding how *LPA* genetic variants contribute to Lp(a) levels in a diverse population. One of the major findings of our study was that there were notably more significant associations between Lp(a) and *LPA* SNPs in non-Hispanic blacks compared to non-Hispanic whites and Mexican Americans. Moreover, nearly half of these associations were exclusive to non-Hispanic blacks. Our results suggest that between-population differences in Lp(a) levels can be explained, in part, by multiple population-specific *cis*-acting variants in *LPA*. While the role of multiple *trans*-acting factors in Lp(a) trait distribution has been disputed[44]–[46] and cannot be ruled out, our results reaffirm the need for more comprehensive studies of the effects of *LPA* variants in large, diverse populations.

## Materials and Methods

### Ethics Statement

All procedures were approved by the CDC Ethics Review Board and written informed consent was obtained from all participants. This candidate gene association study was approved by the CDC Ethics Review Board (protocols #2003-08 and #2006-11) and the University of Washington's Institutional Review Board (IRB #23667; HSRC D committee). Because no identifying information was accessed by the investigators, this study was considered exempt from Human Subjects by Vanderbilt University's Institutional Review Board (IRB #061062; HS2 committee).

### Study Population

Ascertainment of the Third National Health and Nutrition Examination Survey (NHANES III) and method of DNA collection have been previously described[47]–[49] and so will only be briefly described here. The National Health and Nutrition Examination Surveys are cross-sectional surveys conducted by the National Center for Health Statistics (NCHS) at the Centers for Disease Control and Prevention (CDC). NHANES III was conducted between 1988–1990 (phase 1) and 1991–1994 (phase 2)[50], [51]. Like all the NHANES, NHANES III is a complex survey design that over-sampled minorities (non-Hispanic blacks and Mexican Americans), the young, and the elderly. All NHANES have interviews that collect demographic, socioeconomic, dietary, and health-related data. Also, all NHANES study participants undergo a detailed medical examination at a central location known as the Mobile Examination Center (MEC). The medical examination includes the collection of physiological measurements by CDC medical personnel and blood and urine samples for laboratory tests. Beginning with phase 2 of NHANES III, DNA samples were collected from study participants aged 12 years and older.

### Laboratory Measures

Serum total cholesterol, triglycerides, and HDL cholesterol were measured using standard enzymatic methods. LDL cholesterol was calculated using the Friedewald equation, with missing values assigned for samples with triglyceride levels greater than 400 mg/dl. Serum Lp(a) levels were measured immunochemically by enzyme-linked immunosorbant assay (ELISA) (Strategic Diagnostics, Newark, DE), which does not have cross reactivity with plasminogen or LDL and is non-sensitive to apo(a) size heterogeneity[26]. Quality control measures of the Lp(a) assay have been described elsewhere and the reliability of this Lp(a) measurement has been adequately demonstrated [26].

### SNP Selection and Genotyping

Single nucleotide polymorphisms (SNPs) were selected from SeattleSNPs data on European Americans (n = 23) and African Americans (n = 24) re-sequenced for SNP discovery as previously described[25]. Briefly, tagSNPs were chosen for genotyping in both populations separately using LDSelect[52] at minor allele frequency (MAF) >5% and r^2^>0.80. At the time of tagSNPs selection (2006), *LPA* variation data was not available for Mexican Americans or other Hispanic reference samples. Forty-nine SNPs were considered for genotyping, 35 SNPs were targeted for genotyping, and 20 were successfully genotyped. Genotyping was performed using the Illumina GoldenGate assay (as part of a custom 384 OPA) by the Center for Inherited Disease Research (CIDR) through the National Heart Lung and Blood Institute's Resequencing and Genotyping Service. A display of the chromosomal locations of all 20 *LPA* SNPs, along with their relative locations to the 5′ untranslated region (represented by rs1800769) and the kringle repeat (represented by rs9457952 and rs9457986, which flank the kringle repeat), is presented in Supplementary Figure S2.

Genotyping call rates and tests of Hardy Weinberg Equilibrium stratified by self-reported race/ethnicity were calculated for all genotyped *LPA* SNPs (Supplementary Table S1). The average genotyping call rate for all 20 SNPs was 95.9%. SNP rs4073498 was out of Hardy Weinberg Equilibrium (HWE; p<0.01) in all three racial/ethnic groups and was therefore excluded from all analyses as mandated by CDC. Five additional SNPs (rs1321195, rs1652507, rs7755463, rs7450261, and rs41265936) were found to be out HWE in one subpopulation but were carried forward in the analysis. In addition to these quality control metrics, we genotyped blinded duplicates as required by CDC, and all SNPs reported here passed quality control metrics required by CDC. All genotype data reported here were deposited into the NHANES III Genetic database and are available for secondary analysis through CDC.

### Statistical Methods

Analyses were performed for each self-reported race/ethnicity separately. Quality control measures were implemented in PLINK[53]. Tests of association were performed using SAS version 9.1 and were limited to participants greater than 18 years of age who had non-missing Lp(a) levels regardless of fasting status. Each genetic variant was tested for association with ln(Lp(a)+1) levels (a transformation that approximated normality) using linear regression assuming an additive genetic model. Analyses were performed adjusted for age and sex, and results were plotted using Synthesis-View[54], [55]. Data were accessed remotely from the CDC's Research Data Center (RDC) in Hyattsville, Maryland using Analytic Data Research by Email (ANDRE). Statistical significance was defined as p<0.0001, which represents the Bonferroni corrected p-value [p = 0.0008 = 0.05/(20 SNPs × 3 populations)]. Using STATA 10.1, the frequency of risk alleles was compared between populations using Pearson's chi-squared test. Pair-wise linkage disequilibrium (r^2^) was calculated using the Genome Variation Server provided by SeattleSNPs (http://gvs.gs.washington.edu/GVS/). Haplotypes were inferred by SAS/Genetics using the expectation-maximization algorithm in each subpopulation separately.

To account for selection and non-response biases, the National Center for Health Statistics provides a weighting methodology, which has been described elsewhere[56]. We performed tests of association both unweighted (using SAS version 9.1) and weighted (using SUDAAN). The results did not differ appreciably; therefore, unweighted results are presented here and only select weighted results are presented in Supplementary Table S4.

### Genetic Risk Score Calculation

The Genetic Risk Score (GRS) was calculated for every participant, respective to each population separately, using SNPs that were associated with transformed Lp(a) levels at p<0.0001. We used a count method and assumed each SNP to be independently associated with increased levels of Lp(a). Assuming an additive genetic model for each SNP, a value of 2 was given to individuals who were homozygous for the “risk” allele (i.e. the allele associated with increased levels of transformed Lp(a) levels). Values of 1 and 0 were given to genotypes containing 1 or 0 copies of the risk allele, respectively. The GRS was calculated summing the number of risk alleles at each locus. Participants with incomplete genotype data at any SNP used in the GRS were excluded from analysis. Linear regression, with continuous GRS as the independent variable, was used to evaluate the joint effects (R^2^) of associated genetic variants for Lp(a) trait variation. A weighted GRS (WGRS) was also calculated by multiplying each β-coefficient from adjusted tests of association by the number of risk alleles, and then summing the products. Compared to the GRS, the results of the WGRS do not appreciably differ (Supplementary Table S5); therefore, GRS was used for the main analyses in the paper.

## Supporting Information

Figure S1 **Pair-wise linkage disequilibrium (r^2^) calculated for 19** ***LPA*** **SNPs in non-Hispanic whites (A), non-Hispanic blacks (B), and Mexican Americans (C) in NHANES III.**
(DOC)

Figure S2 **Location of genotyped** ***LPA*** **SNPs relative to the kringle repeat region and a SNP in the 5′ untranslated region.** Synthesis-View[1] was used to plot the 20 *LPA* SNPs genotyped in this study. Three other SNPs not genotyped in this study are also represented in this plot within the boxes: rs1800769 (which represents a 5′ UTR SNP genotyped by Rainwater et al 1997[2]) and rs9457986 and rs9457952, which flank the kringle repeat. Chromosomal locations are based on genome build 36.
(DOC)

Table S1 **SNP location and genotyping quality control metrics, stratified by race/ethnicity.**
(DOC)

Table S2 **Frequency of** ***LPA*** **variants in HapMap samples.**
(DOC)

Table S3 ***LPA*** **common haplotypes and haplotype frequencies.** Only haplotypes with frequencies >5% in at least one population are displayed. Alleles are ordered based on chromosomal location (5′ to 3′). Frequencies >5% are in bold.
(DOC)

Table S4 **Associations between** ***LPA*** **SNPs and Lp(a) levels, weighted for selection and non-response biases.** The association of *LPA* SNPs with log transformed Lp(a) levels is shown by a regression coefficient (beta, β) and 95% confidence interval (CI) for each SNP, adjusted for age and sex. Measures of variance explained (R^2^) are provided for each SNP based on unadjusted regressions. Significant associations (P-value<0.0001) are in bold.
(DOC)

Table S5 **Additive effects of** ***LPA*** **alleles associated with increased Lp(a) levels.** The amount of variance explained (R^2^) in transformed Lp(a) levels by the Weighted Genetic Risk Score (WGRS) is displayed, along with the median WGRS score, WGRS interquartile range (IQR), regression coefficient (beta, β) and 95% confidence interval (CI) for each association.
(DOC)
