# Supplementary material for: Variation in LPA Is Associated with Lp(a) Levels in Three Populations from the Third National Health and Nutrition Examination Survey
Source: PLoS One. 2011 Jan 28;6(1):e16604. doi: 10.1371/journal.pone.0016604 (PMC3030597; doi:10.1371/journal.pone.0016604)
Supplement: Figure S1 — Pair-wise linkage disequilibrium (r2) calculated for 19 LPA SNPs in non-Hispanic whites (A), non-Hispanic blacks (B), and Mexican Americans (C) in NHANES III. (DOC) [file pone.0016604.s001.doc]

**Figure S1. Pair-wise linkage disequilibrium (r^2^) calculated for 19 *LPA* SNPs in non-Hispanic whites (A), non-Hispanic blacks (B), and Mexican Americans (C) in NHANES III.**

**
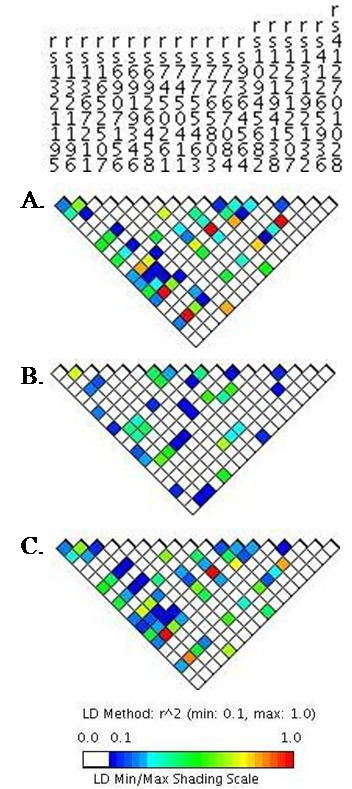
**
