# Supplementary material for: Variation in LPA Is Associated with Lp(a) Levels in Three Populations from the Third National Health and Nutrition Examination Survey
Source: PLoS One. 2011 Jan 28;6(1):e16604. doi: 10.1371/journal.pone.0016604 (PMC3030597; doi:10.1371/journal.pone.0016604)
Supplement: Figure S2 — Location of genotyped LPA SNPs relative to the kringle repeat region and a SNP in the 5′ untranslated region. Synthesis-View[1] was used to plot the 20 LPA SNPs genotyped in this study. Three other SNPs not genotyped in this study are also represented in this plot within the boxes: rs1800769 (which represents a 5′ UTR SNP genotyped by Rainwater et al 1997[2]) and rs9457986 and rs9457952, which flank the kringle repeat. Chromosomal locations are based on genome build 36. (DOC) [file pone.0016604.s002.doc]

**Figure S2. Location of genotyped *LPA* SNPs relative to the kringle repeat region and a SNP in the 5′ untranslated region.** Synthesis-View[1] was used to plot the 20 *LPA* SNPs genotyped in this study. Three other SNPs not genotyped in this study are also represented in this plot within the boxes: rs1800769 (which represents a 5′ UTR SNP genotyped by Rainwater et al 1997[2]) and rs9457986 and rs9457952, which flank the kringle repeat. Chromosomal locations are based on genome build 36.


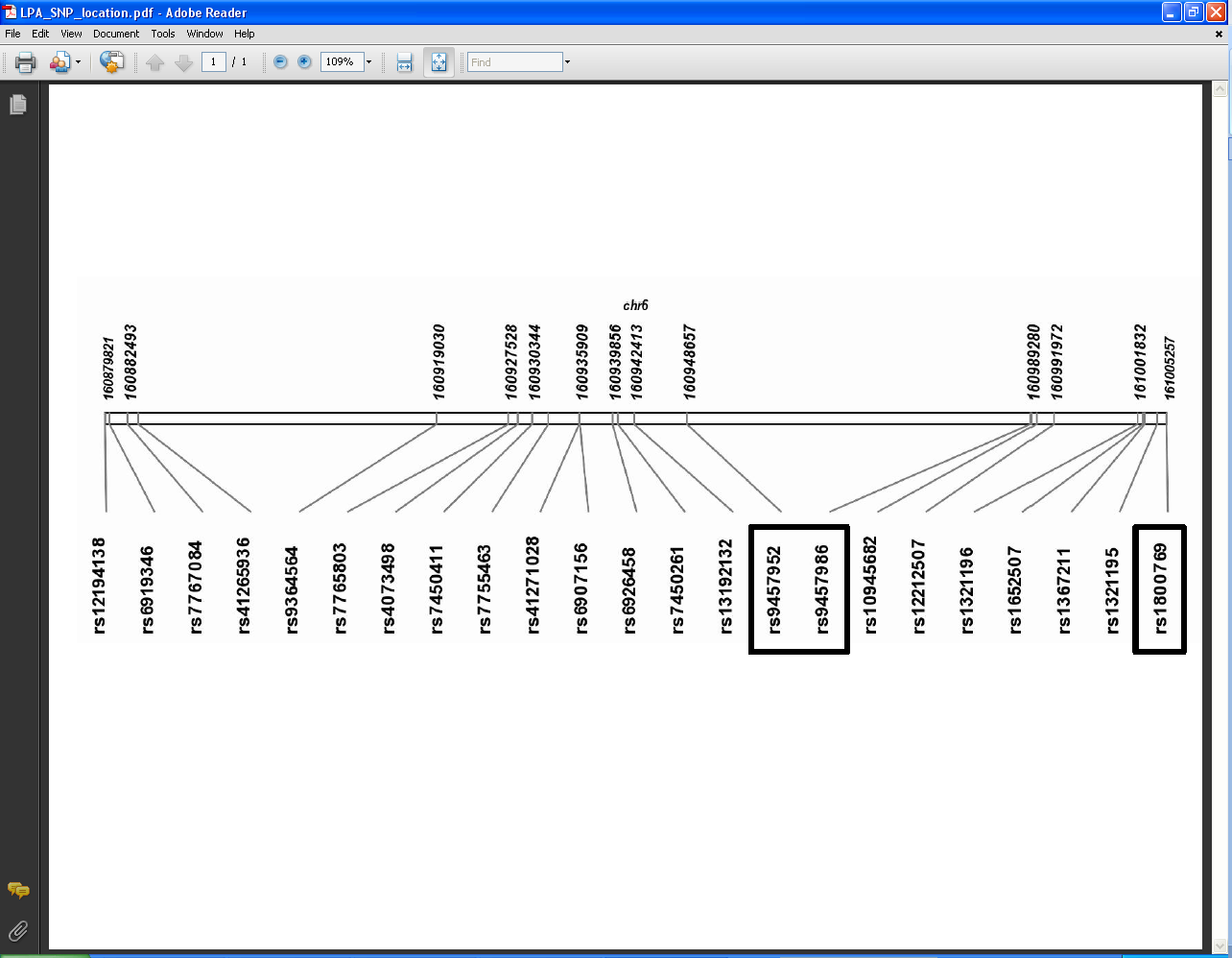


Reference List

1. Pendergrass S, Dudek S, Crawford D, Ritchie M (2010) Synthesis-View: visualization and interpretation of SNP association results for multi-cohort, multi-phenotype data and meta-analysis. BioData Mining. In press.

2. Rainwater DL, Kammerer CM, Vandeberg JL, Hixson JE (1997) Characterization of the genetic elements controlling lipoprotein(a) concentrations in Mexican Americans. Evidence for at least three controlling elements linked to LPA, the locus encoding apolipoprotein(a). Atherosclerosis 128: 223-233.
