# Supplementary material for: Variation in LPA Is Associated with Lp(a) Levels in Three Populations from the Third National Health and Nutrition Examination Survey
Source: PLoS One. 2011 Jan 28;6(1):e16604. doi: 10.1371/journal.pone.0016604 (PMC3030597; doi:10.1371/journal.pone.0016604)
Supplement: Table S1 — SNP location and genotyping quality control metrics, stratified by race/ethnicity. (DOC) [file pone.0016604.s003.doc]

Table S1. SNP location and genotyping quality control metrics, stratified by race/ethnicity.

| **SNP** | **Position (bp)**  (Build 36) | **Location** | **Alleles^‡^** | **Non-Hispanic Whites**  n=2,631 | | | | **Non-Hispanic Blacks**  n=2,108 | | | | **Mexican Americans**  n=2,073 | | | |
| --- | --- | --- | --- | --- | --- | --- | --- | --- | --- | --- | --- | --- | --- | --- | --- |
|  |  |  |  | **G.E. %** | **HWE** | **MA** | **MAF** | **G.E. %** | **HWE** | **MA** | **MAF** | **G.E.%** | **HWE** | **MA** | **MAF** |
| **rs1321196** | 161001832 | intron | A/G | 96.0 | 0.488 | G | 0.358 | 96.1 | 0.527 | G | 0.436 | 96.0 | 0.910 | G | 0.272 |
| **rs1321195** | 161004146 | intron | A/G | 95.8 | 0.793 | A | 0.131 | 96.3 | 0.695 | A | 0.030 | 95.8 | 4.3E-4 | A | 0.089 |
| **rs1367211** | 161002685 | intron | A/G | 95.8 | 0.249 | A | 0.274 | 95.8 | 1.000 | A | 0.480 | 95.8 | 0.463 | A | 0.214 |
| **rs4073498** | 160928635 | intron | A/G | 94.2 | 4.0E-22 | A | 0.368 | 92.3 | 0.009 | G | 0.452 | 94.2 | 0.005 | A | 0.272 |
| **rs1652507** | 161002451 | intron | A/G | 95.5 | 0.101 | G | 0.168 | 96.1 | 0.760 | G | 0.078 | 95.5 | 0.002 | G | 0.428 |
| **rs6907156** | 160935999 | intron | A/G | 96.2 | 1.000 | G | 0.003 | 96.1 | 0.141 | G | 0.162 | 96.2 | 1.000 | G | 0.019 |
| **rs6919346** | 160880349 | intron | A/G | 95.4 | 0.038 | A | 0.175 | 96.3 | 1.000 | A | 0.035 | 95.4 | 0.895 | A | 0.094 |
| **rs6926458** | 160939856 | intron | A/G | 96.0 | 0.352 | G | 0.219 | 96.3 | 0.557 | G | 0.106 | 96.0 | 0.395 | G | 0.156 |
| **rs7755463** | 160932260 | intron | A/G | 96.3 | 0.001 | A | 0.005 | 96.1 | 0.623 | A | 0.345 | 96.3 | 0.664 | A | 0.028 |
| **rs7767084** | 160882493 | intron | A/G | 96.0 | 0.715 | G | 0.163 | 96.4 | 0.510 | G | 0.034 | 96.0 | 0.789 | G | 0.147 |
| **rs9364564** | 160919030 | intron | A/G | 95.8 | 0.683 | A | 0.178 | 96.3 | 0.397 | A | 0.086 | 95.8 | 0.403 | A | 0.140 |
| **rs12212507** | 160991972 | intron | A/G | 96.1 | 0.246 | A | 0.055 | 96.4 | 1.000 | A | 0.007 | 96.1 | 1.000 | A | 0.012 |
| **rs13192132** | 160942413 | intron | A/G | 95.8 | 0.513 | G | 0.353 | 96.3 | 0.858 | G | 0.145 | 95.8 | 0.504 | G | 0.245 |
| **rs10945682** | 160989931 | intron | A/G | 95.6 | 0.279 | A | 0.359 | 96.2 | 0.223 | G | 0.438 | 95.6 | 1.000 | A | 0.279 |
| **rs12194138** | 160879821 | intron | A/T | 96.0 | 0.776 | T | 0.168 | 96.4 | 1.000 | T | 0.027 | 96.0 | 0.486 | T | 0.069 |
| **rs7450261** | 160940495 | intron | A/G | 96.2 | 1.000 | A | 0.001 | 96.0 | 0.006 | A | 0.053 | 96.2 | 1.000 | A | 0.002 |
| **rs7450411** | 160930344 | intron | A/C | 95.6 | 0.734 | A | 0.179 | 96.1 | 0.637 | A | 0.136 | 95.6 | 0.311 | A | 0.142 |
| **rs7765803** | 160927528 | L1358V | C/G | 95.7 | 0.589 | C | 0.331 | 95.9 | 0.823 | G | 0.457 | 95.7 | 0.729 | C | 0.264 |
| **rs41265936** | 160883764 | G1822A | C/G | 96.2 | 1.000 | G | 0.001 | 96.0 | 4.1E-4 | G | 0.062 | 96.2 | 1.000 | G | 0.006 |
| **rs41271028** | 160935909 | intron | A/T | 96.2 | 1.000 | T | 0.003 | 96.4 | 0.150 | T | 0.094 | 96.2 | 1.000 | T | 0.013 |

^‡^Referent allele listed first

Abbreviations: Base pair (bp), genotyping efficiency (GE), Hardy Weinberg Equilibrium (HWE), minor allele (MA), minor allele frequency (MAF).
