# Supplementary material for: Variation in LPA Is Associated with Lp(a) Levels in Three Populations from the Third National Health and Nutrition Examination Survey
Source: PLoS One. 2011 Jan 28;6(1):e16604. doi: 10.1371/journal.pone.0016604 (PMC3030597; doi:10.1371/journal.pone.0016604)
Supplement: Table S2 — Frequency of LPA variants in HapMap samples. (DOC) [file pone.0016604.s004.doc]

**Table S2. Frequency of *LPA*** variants in HapMap samples.

| **SNP** | **Alleles** | **CEU**  **n=60** | | **YRI**  **n=60** | | **HCB**  **n=45** | | **JPT**  **n=45** | | **ASW**  **n=47** | | **MEX**  **n=47** | |
| --- | --- | --- | --- | --- | --- | --- | --- | --- | --- | --- | --- | --- | --- |
|  |  | **MA** | **MAF** | **MA** | **MAF** | **MA** | **MAF** | **MA** | **MAF** | **MA** | **MAF** | **MA** | **MAF** |
| rs1321196 | C/T | C | 0.38 | C | 0.43 | T | 0.49 | C | 0.49 | C | 0.47 | C | 0.26 |
| rs1321195 | A/G | A | 0.17 | mono | 0.00 | A | 0.22 | A | 0.28 | N/A | N/A | N/A | N/A |
| rs1367211 | T/C | T | 0.30 | C | 0.46 | T | 0.19 | T | 0.29 | T | 0.50 | T | 0.17 |
| rs1652507 | C/T | C | 0.13 | C | 0.06 | C | 0.38 | C | 0.38 | C | 0.06 | C | 0.41 |
| rs6907156 | C/T | C | 0.01 | N/A | N/A | mono | 0.00 | mono | 0.00 | N/A | N/A | N/A | N/A |
| rs6919346 | T/C | T | 0.15 | T | 0.01 | mono | 0.00 | mono | 0.00 | T | 0.06 | T | 0.16 |
| rs6926458 | G/A | G | 0.25 | G | 0.10 | A | 0.49 | G | 0.48 | G | 0.16 | G | 0.16 |
| rs7755463 | T/C | T | 0.01 | T | 0.40 | mono | 0.00 | mono | 0.00 | T | 0.30 | N/A | N/A |
| rs7767084 | C/T | C | 0.14 | mono | 0.00 | C | 0.26 | C | 0.31 | C | 0.03 | C | 0.09 |
| rs10945682 | A/G | A | 0.38 | G | 0.38 | G | 0.49 | A | 0.50 | G | 0.49 | A | 0.26 |
| rs7450261 | C/T | mono | 0.00 | T | 0.08 | mono | 0.00 | mono | 0.00 | T | 0.05 | N/A | N/A |
| rs7765803 | C/G | C | 0.33 | G | 0.40 | G | 0.50 | G | 0.48 | G | 0.48 | C | 0.22 |

Abbreviations: Utah residents with Northern and Western European ancestry (CEU); Yoruba in Ibadan, Nigeria (YRI); Han Chinese in Beijing, China (HCB); Japanese in Tokyo, Japan (JPT); African ancestry in Southwest USA (ASW); Mexican ancestry in Los Angeles, California (MEX); minor allele (MA); minor allele frequency (MAF); monomorphic (mono); SNPs designated not applicable (N/A) were not genotyped in HapMap in that particular population.
