# Supplementary material for: Variation in LPA Is Associated with Lp(a) Levels in Three Populations from the Third National Health and Nutrition Examination Survey
Source: PLoS One. 2011 Jan 28;6(1):e16604. doi: 10.1371/journal.pone.0016604 (PMC3030597; doi:10.1371/journal.pone.0016604)
Supplement: Table S3 — LPA common haplotypes and haplotype frequencies. Only haplotypes with frequencies >5% in at least one population are displayed. Alleles are ordered based on chromosomal location (5′ to 3′). Frequencies >5% are in bold. (DOC) [file pone.0016604.s005.doc]

**Table S3. *LPA* common haplotypes and haplotype frequencies.** Only haplotypes with frequencies > 5% in at least one population are displayed.

| **Haplotype Number** | **Haplotype Alleles** | **Frequency in**  **Non-Hispanic Whites** | **Frequency in**  **Non-Hispanic Blacks** | **Frequency in Mexican Americans** |
| --- | --- | --- | --- | --- |
| 1 | A-C-T-C-G-G-C-C-A-T-A-C-T-G-G-A-G-G-C | **0.076** | **0.062** | **0.391** |
| 2 | A-C-T-C-G-G-C-C-A-T-A-C-T-G-G-A-A-G-C | **0.207** | **0.253** | **0.142** |
| 3 | A-T-T-C-G-G-C-C-A-T-A-C-T-G-G-A-A-G-C | **0.151** | 0.030 | **0.078** |
| 4 | A-C-C-C-A-C-A-C-A-T-G-C-C-A-G-G-A-A-T | **0.088** | 0.016 | **0.058** |
| 5 | A-C-T-C-G-C-C-C-A-T-A-C-C-A-G-G-A-A-C | **0.107** | 0.018 | **0.057** |
| 6 | T-C-T-C-G-G-C-C-A-T-A-C-T-G-G-A-A-G-C | **0.086** | 0.012 | 0.040 |
| 7 | A-C-T-C-G-C-C-T-T-C-A-C-T-A-G-G-A-A-C | 0.002 | **0.091** | 0.013 |
| 8 | A-C-T-C-G-C-C-T-A-T-A-C-T-A-G-G-A-A-C | 0.001 | **0.151** | 0.007 |

Alleles are ordered based on chromosomal location (5' to 3'). Frequencies > 5% are in bold.
