# Supplementary material for: Variation in LPA Is Associated with Lp(a) Levels in Three Populations from the Third National Health and Nutrition Examination Survey
Source: PLoS One. 2011 Jan 28;6(1):e16604. doi: 10.1371/journal.pone.0016604 (PMC3030597; doi:10.1371/journal.pone.0016604)
Supplement: Table S4 — Associations between LPA SNPs and Lp(a) levels, weighted for selection and non-response biases. The association of LPA SNPs with log transformed Lp(a) levels is shown by a regression coefficient (beta, β) and 95% confidence interval (CI) for each SNP, adjusted for age and sex. Measures of variance explained (R2) are provided for each SNP based on unadjusted regressions. Significant associations (P-value<0.0001) are in bold. (DOC) [file pone.0016604.s006.doc]

**Table S4. Associations between *LPA* SNPs and Lp(a) levels, weighted for selection and non-response biases.** The association of *LPA* SNPs with log transformed Lp(a) levels is shown by a regression coefficient (beta, β) and 95% confidence interval (CI) for each SNP, adjusted for age and sex. Measures of variance explained (R^2^) are provided for each SNP based on unadjusted regressions. Significant associations (P-value < 0.0001) are in bold.

| **SNPs** | **Non-Hispanic Whites**  n=2,397 | | | **Non-Hispanic Blacks**  n=1,711 | | | **Mexican Americans**  n=1,749 | | |
| --- | --- | --- | --- | --- | --- | --- | --- | --- | --- |
|  | β  (95% CI) | R^2^ | P-value | β  (95% CI) | R^2^ | P-value | β  (95% CI) | R^2^ | P-value |
| **rs1321196** | -0.17  (-0.28, -0.06) | 0.0061 | 0.0043 | 0.19  (0.09, 0.29) | 0.0171 | 0.0005 | 0.18  (0.07, 0.28) | 0.0069 | 0.0018 |
| **rs1321195** | 0.10  (-0.01, 0.21) | 0.0012 | 0.0651 | 0.49  (0.16, 0.81) | 0.0108 | 0.0055 | 0.38  (0.16, 0.60) | 0.0114 | 0.0015 |
| **rs1367211** | 0.02  (-0.09, 0.12) | 0.0000 | 0.74271 | -0.24  (-0.36, -0.12) | 0.0271 | 0.0005 | -0.19  (-0.30, -0.08) | 0.0068 | 0.0016 |
| **rs1652507** | -0.04  (-0.17, 0.10) | 0.0002 | 0.6083 | -0.44  (-0.62, -0.26) | 0.0225 | **3.44x10^-5^** | -0.58  (-0.69, -0.47) | 0.0977 | **< 2x10^-10^** |
| **rs6907156** | 0.03  (-0.82, 0.88) | 0.0000 | 0.9404 | 0.14  (0.04, 0.24) | 0.0047 | 0.0111 | 0.67  (0.34, 1.00) | 0.0088 | 0.0003 |
| **rs6919346** | 0.51  (0.36, 0.66) | 0.0418 | **2.23x10^-7^** | 0.77  (0.62, 0.91) | 0.0381 | **< 2x10^-10^** | 0.21  (-0.02, 0.44) | 0.0040 | 0.0762 |
| **rs6926458** | -0.29  (-0.42, -0.15) | 0.0145 | 0.0002 | -0.44  (-0.60, -0.28) | 0.0325 | **1.01x10^-5^** | -0.11  (-0.24, 0.02) | 0.0016 | 0.0860 |
| **rs7755463** | -0.40  (-1.04, 0.24) | 0.0007 | 0.2088 | -0.33  (-0.41, -0.24) | 0.0457 | **4.59 x10^-8^** | -0.89  (-1.08, -0.71) | 0.0251 | **< 7x10^-10^** |
| **rs7767084** | 0.01  (-0.08, 0.11) | 0.0000 | 0.8006 | -0.39  (-0.67, -0.11) | 0.0086 | 0.0089 | 0.08  (-0.05, 0.21) | 0.0010 | 0.2143 |
| **rs9364564** | 0.24  (0.08, 0.39) | 0.0084 | 0.0044 | 0.33  (0.20, 0.46) | 0.0161 | **2.00x10^-5^** | 0.05  (-0.09, 0.20) | 0.0003 | 0.4614 |
| **rs12212507** | 0.03  (-0.15, 0.22) | 0.0001 | 0.6958 | 0.71  (0.13, 1.28) | 0.0074 | 0.0179 | 0.55  (0.18, 0.92) | 0.0030 | 0.0057 |
| **rs13192132** | -0.18  (-0.28, -0.08) | 0.0067 | 0.0013 | -0.43  (-0.54, -0.32) | 0.0408 | **3.90x10^-8^** | 0.06  (-0.06, 0.18) | 0.0007 | 0.3151 |
| **rs10945682** | 0.16  (0.05, 0.27) | 0.0055 | 0.0048 | -0.13  (-0.21, -0.05) | 0.0079 | 0.0019 | -0.22  (-0.33, -0.11) | 0.0106 | 0.0005 |
| **rs12194138** | -0.43  (-0.54, -0.32) | 0.0255 | **3.52x10^-8^** | 0.30  (-0.05, 0.64) | 0.0045 | 0.0871 | -0.49  (-0.69, -0.29) | 0.0155 | **4.53x10^-5^** |
| **rs7450261** | -0.49  (-2.52, 1.54) | 0.0002 | 0.6209 | 0.31  (0.15, 0.48) | 0.0087 | 0.0007 | -0.03  (-1.92, 1.87) | 0.0000 | 0.97843 |
| **rs7450411** | 0.24  (0.09, 0.4) | 0.0088 | 0.0033 | 0.37  (0.27, 0.47) | 0.0308 | **1.69x10^-7^** | 0.06  (-0.07, 0.20) | 0.0005 | 0.3555 |
| **rs7765803** | -0.08  (-0.2, 0.03) | 0.0013 | 0.1436 | 0.12  (0.01, 0.23) | 0.0064 | 0.0302 | 0.26  (0.15, 0.38) | 0.0152 | **9.09x10^-5^** |
| **rs41265936** | -1.11  (-1.61, -0.62) | 0.0020 | 0.0001 | -0.18  (-0.33, -0.02) | 0.0032 | 0.0280 | -0.82  (-1.22, -0.41) | 0.0043 | 0.0004 |
| **rs41271028** | -0.39  (-0.92, 0.14) | 0.0003 | 0.1421 | -0.05  (-0.15, 0.06) | 0.0005 | 0.3700 | -0.58  (-0.95, -0.22) | 0.0049 | 0.0031 |
