# Supplementary material for: Variation in LPA Is Associated with Lp(a) Levels in Three Populations from the Third National Health and Nutrition Examination Survey
Source: PLoS One. 2011 Jan 28;6(1):e16604. doi: 10.1371/journal.pone.0016604 (PMC3030597; doi:10.1371/journal.pone.0016604)
Supplement: Table S5 — Additive effects of LPA alleles associated with increased Lp(a) levels. The amount of variance explained (R2) in transformed Lp(a) levels by the Weighted Genetic Risk Score (WGRS) is displayed, along with the median WGRS score, WGRS interquartile range (IQR), regression coefficient (beta, β) and 95% confidence interval (CI) for each association. (DOC) [file pone.0016604.s007.doc]

**Table S5. Additive effects of *LPA* alleles associated with increased Lp(a) levels.** The amount of variance explained (R^2^) in transformed Lp(a) levels by the Weighted Genetic Risk Score (WGRS) is displayed, along with the median WGRS score, WGRS interquartile range (IQR), regression coefficient (beta, β) and 95% confidence interval (CI) for each association.

|  | **Non-Hispanic Whites** | **Non-Hispanic Blacks** | **Mexican Americans** |
| --- | --- | --- | --- |
| Total n | 2269 | 1605 | 1665 |
| No. SNPs used in WGRS | 3 | 12 | 6 |
| Median WGRS (IQR) | 1.50 (0.45) | 8.24 (1.01) | 1.56 (0.53) |
| β (95% CI) | 0.87 (0.75-0.99) | 0.33 (0.28-0.38) | 0.87 (0.75-0.99) |
| P-value | <10^-40^ | <10^-38^ | <10^-47^ |
| R^2^ | 0.08 | 0.10 | 0.12 |
